# Dependence of PINK1 accumulation on mitochondrial redox system

**DOI:** 10.1111/acel.13211

**Published:** 2020-08-11

**Authors:** Feng Gao, Yan Zhang, Xiaoou Hou, Zhouteng Tao, Haigang Ren, Guanghui Wang

**Affiliations:** ^1^ Laboratory of Molecular Neuropathology Jiangsu Key laboratory of Neuropsychiatric Disorders & Department of Pharmacology College of Pharmaceutical Sciences Soochow University Suzhou Jiangsu China; ^2^ Neurodegenerative Disorder Research Center, Division of Life Sciences and Medicine University of Science and Technology of China Hefei Anhui China; ^3^ Center for Drug Safety Evaluation and Research State Key Laboratory of New Drug Research Shanghai Institute of Materia Medica Chinese Academy of Sciences Shanghai China

**Keywords:** autophagy, mitochondrion, mitophagy, neurodegenerative diseases, PINK1

## Abstract

Accumulation of PINK1 on the outer mitochondrial membrane (OMM) is necessary for PINK‐mediated mitophagy. The proton ionophores, like carbonyl cyanide m‐chlorophenylhydrazone (CCCP) and carbonyl cyanide‐4‐(trifluoromethoxy)phenylhydrazone (FCCP), inhibit PINK1 import into mitochondrial matrix and induce PINK1 OMM accumulation. Here, we show that the CHCHD4/GFER disulfide relay system in the mitochondrial intermembrane space (IMS) is required for PINK1 stabilization when mitochondrial membrane potential is lost. Activation of CHCHD4/GFER system by mitochondrial oxidative stress or inhibition of CHCHD4/GFER system with antioxidants can promote or suppress PINK1 accumulation, respectively. Thus data suggest a pivotal role of CHCHD4/GFER system in PINK1 accumulation. The amyotrophic lateral sclerosis‐related superoxide dismutase 1 mutants dysregulated redox state and CHCHD4/GFER system in the IMS, leading to inhibitions of PINK1 accumulation and mitophagy. Thus, the redox system in the IMS is involved in PINK1 accumulation and damaged mitochondrial clearance, which may play roles in mitochondrial dysfunction‐related neurodegenerative diseases.

## Graphical abstract

PINK1 is accumulated on the depolarized mitochondria dependent on the disulfide relay system in the IMS. Unfolded PINK1 enters the IMS through the TOM and interacts with oxidized CHCHD4 to avoid releasing to cytosol from the membrane potential lost mitochondria. The PINK1 in the TOM complex is further transported into the OMM through some proteins in the TOM.

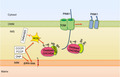

## 1 INTRODUCTION

Parkinson’s disease (PD)‐associated gene products, like Parkin and PTEN‐induced putative kinase 1 (PINK1), have been suggested to be the key components in mitochondrial quality control system (Narendra et al., 2010; Park et al., 2006). Induction of mitochondrial depolarization by a mitochondrial uncoupler carbonyl cyanide m‐chlorophenylhydrazone (CCCP) or carbonyl cyanide‐4‐(trifluoromethoxy)phenylhydrazone (FCCP) results in an accumulation of PINK1 on the outer mitochondrial membrane (OMM), which induces Parkin selectively recruitment to damaged mitochondria to drive mitochondrial degradation (Chen & Dorn, 2013; Gao et al., 2015; Geisler et al., 2010; Narendra, Tanaka, Suen, & Youle, 2008; Shaid, Brandts, Serve, & Dikic, 2013). The mitochondrial accumulated PINK1 phosphorylates both Parkin and ubiquitin, inducing Parkin E3 ligase activity and translocation to mitochondria (Koyano et al., 2014; Ordureau et al., 2015; Shiba‐Fukushima et al., 2014; Wauer et al., 2015). Activation of Parkin induces the OMM protein ubiquitination for autophagy adaptor‐mediated mitophagy (Shaid et al., 2013).

In healthy mitochondria with normal membrane potential (ΔΨm), PINK1 is continuously imported to the inner mitochondrial membrane (IMM) through the translocase of the outer membrane (TOM) and TIM23 complexes (Greene et al., 2012; Jin et al., 2010; Lazarou, Jin, Kane, & Youle, 2012). The N‐terminal mitochondrial targeting signal (MTS) of PINK1 is removed by mitochondrial processing peptidase (MPP) in matrix, and PINK1 is then further cleaved by rhomboid family protease presenilin‐associated rhomboid‐like protein (PARL) at Ala103 that spans in the IMM in a ΔΨm dependent manner. The cleaved/processed PINK1 is released into the cytosol from the TOM complex and degraded by the proteasome (Deas et al., 2011; Greene et al., 2012; Yamano & Youle, 2013). In damaged mitochondria, loss of ΔΨm leads to a failure of PINK1 importing to the IMM, which causes accumulation of unprocessed PINK1 on the OMM (Narendra et al., 2010). Thus, loss of ΔΨm is associated with PINK1 accumulation on the OMM.

Mitochondrial proteins are initially imported to mitochondria through the TOM complex, and then transported to the OMM, the intermembrane space (IMS) and the IMM through different pathways based on their specific signals (Chacinska, Koehler, Milenkovic, Lithgow, & Pfanner, 2009; Schmidt, Pfanner, & Meisinger, 2010). Due to the size of the protein import channels on mitochondrial membrane, proteins must be unfolded to pass through the channels (Schwartz & Matouschek, 1999) to a specific site in/on mitochondria (Matouschek, Pfanner, & Voos, 2000). It can be expected that with normal ΔΨm, the processed PINK1 keeps unfolding at the C‐terminus so that it is able to slide back to the cytosol through the TOM. However, it is largely unknown how the unprocessed PINK1 escapes from its release to the cytosol and stabilizes on the OMM after passing through the TOM at unfolding status when ΔΨm is lost.

Here, we show that the machineries for mitochondrial protein transport play roles in PINK1 stabilization. Under a mitochondrial oxidative status, the CHCHD4 in the IMS interacts with PINK1 to facilitate the stability of PINK1 on the OMM.

## 2 RESULTS

### 2.1 ΔΨm is necessary but not sufficient for mitophagy induction

**Figure 1:**
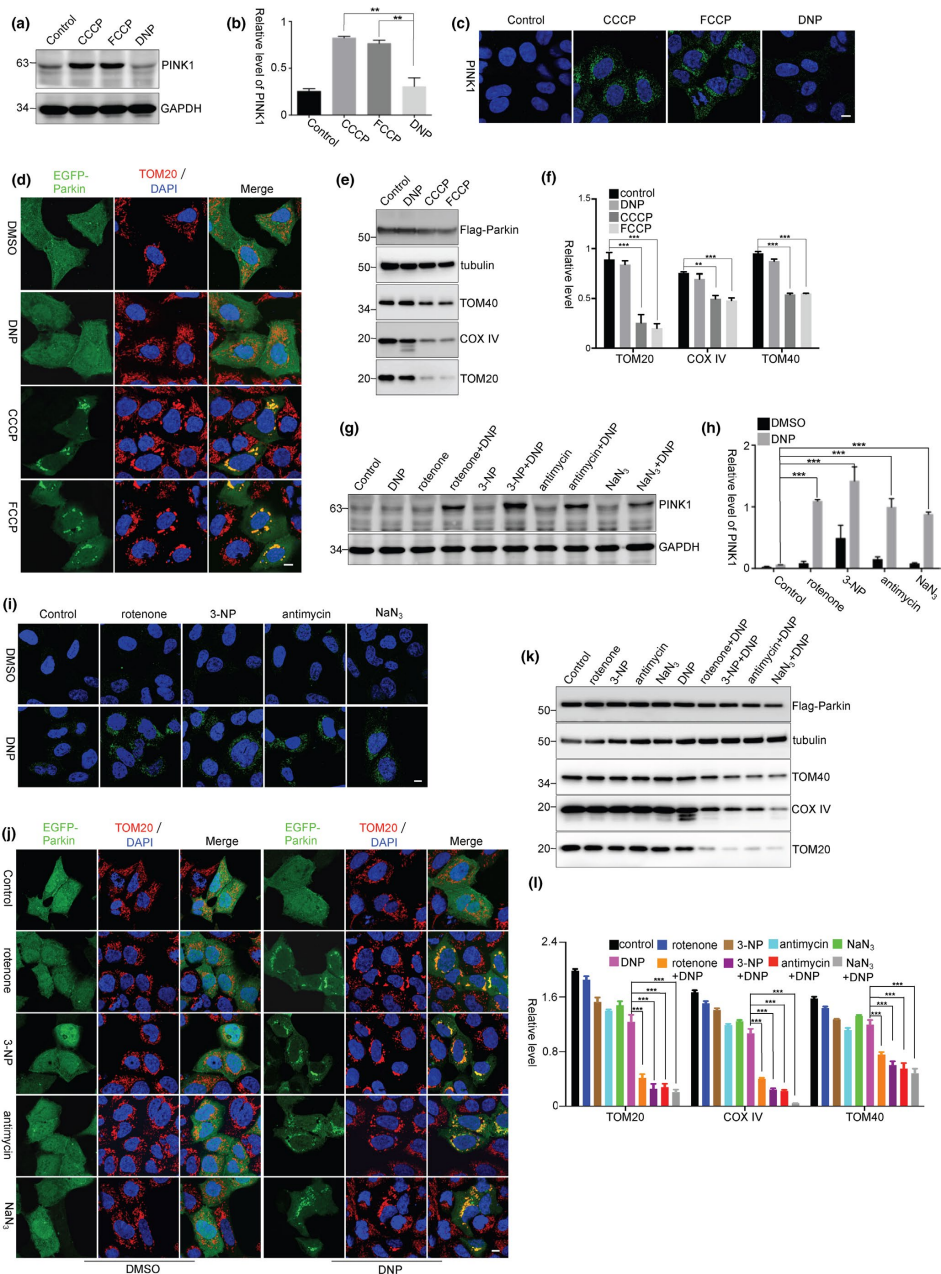
PINK1 accumulation requires mitochondrial oxidative stress. (a ,b) HEK293 cells were treated with CCCP (5 μM), FCCP (5 μM), or DNP (0.5 mM) for 3 hr. The relative levels of PINK1 to GAPDH from three independent experiments were shown in (b). Values are represented as the mean ± *SEM*. **p < 0.01 by one‐way ANOVA. (c) HEK293 cells were treated as a. The cells were subject to immunocytochemical staining with PINK1 antibody (green). The nuclei were stained with DAPI (blue). Scale bar, 10 μm. (d) HEK293 cells were transfected with EGFP‐Parkin and treated with CCCP (5 μM), FCCP (5 μM), or DNP (0.5 mM) for 3 hr. The cells were then subjected to immunocytochemical staining with TOM20 antibody (red). Scale bar, 10 μm. (e, f) HEK293 cells were transfected with Flag‐Parkin and treated with CCCP (5 μM), FCCP (5 μM), or DNP (0.5 mM) for 24 hr. The relative levels of TOM20, TOM40, and COX IV to tubulin from three independent experiments were shown in (f). Mean ± *SEM*, **p < 0.01, ***p < 0.001 by one‐way ANOVA. (g, h) HEK293 cells were treated with rotenone (1 μM), 3‐NP (10 mM), antimycin (10 μM), or NaN_3_ (5 mM) for 2 hr, followed by DNP treatment for 3 hr. The relative levels of PINK1 to GAPDH from three independent experiments were quantified (h). Mean ±SEM, *** *p* < 0.001 by two‐way ANOVA. (i) HEK293 cells were treated as g. The cells were subjected to immunocytochemical staining with PINK1 antibody (green). Scale bar, 10 μm. (j) HEK293 cells were transfected with EGFP‐Parkin and treated with rotenone (1 μM), 3‐NP (10 mM), antimycin (10 μM), or NaN_3_ (5 mM) for 2 hr, followed by DNP treatment for 3 hr. The cells were then subjected to immunocytochemical staining with TOM20 antibody (red). Scale bar, 10 μm. (k, l) HEK293 cells were transfected with Flag‐Parkin and treated with rotenone (1 μM), 3‐NP (10 mM), antimycin (10 μM), or NaN_3_ (5 mM) for 2 hr, followed by DNP treatment for 24 hr. The relative levels of TOM20, TOM40, and COX IV to tubulin from three independent experiments were quantified (l). Mean ± *SEM*, ***p < 0.001 by one‐way ANOVA

Loss of ΔΨm results in an accumulation of PINK1 on the OMM (Geisler et al., 2010; Jin et al., 2010; Narendra et al., 2010). In our observations, 2,4‐dinitrophenol (DNP) effectively abrogated ΔΨm as FCCP or CCCP did (Figure S1a); however, it failed to induce PINK1 accumulation (Figure 1a,b) or enrichment on mitochondria (Figure 1c). In addition, CCCP and FCCP induced Parkin recruitment onto mitochondria (Figure 1d) and resulted in mitophagy, indicated by decreases of mitochondrial components TOM40, COXIV, and TOM20 (Figure 1e,f). However, DNP failed to induce mitochondrial recruitment of EGFP‐Parkin (Figure 1d) or mitochondrial degradation (Figure 1e,f), suggesting that there is other factor(s) involved in the full‐length PINK1 accumulation besides loss of ΔΨm.

A low dose of proton ionophore treatment (DNP 2 μM or FCCP 5 nM) has been suggested to reduce reactive oxygen species (ROS) production in cells (Kuznetsov et al., 2008), but a high dose (20 μM) of FCCP or CCCP treatment quickly induces ROS generation (Izeradjene, Douglas, Tillman, Delaney, & Houghton, 2005; Wang, Li, et al., 2014). In HEK293 cells, we observed that CCCP (5 μM) or FCCP (5 μM) robustly induced mitochondrial ROS and superoxide generation, but DNP, even at a concentration of 0.5 mM, did not (Figure S1b,c). It has been reported that cells do not change ΔΨm when they are shortly exposed to a low dose of rotenone, a mitochondrial complex I inhibitor (Ward, Rego, Frenguelli, & Nicholls, 2000). Consistently, a short‐time treatment with a low dose of rotenone, mitochondrial complex II inhibitor (3‐NP), complex III inhibitor (antimycin), or complex IV inhibitor (NaN_3_) did not significantly affect ΔΨm (Figure S2a) but induced a significant superoxide generation in mitochondria (Figure S2b,c). Mitochondrial respiratory chain inhibitors also did not affect PINK1 processing and degradation, as the processed/cleaved PINK1 was still presented after the cells were treated with the proteasome inhibitor MG132 (Figure S2d). Thus, mitochondrial respiratory chain inhibitors do not significantly affect ΔΨm, but induce ROS generation. Interestingly, DNP induced PINK1 accumulation and Parkin‐mediated mitophagy in cells that were pretreated with those mitochondrial inhibitors (Figure 1g–l). In contrast to mitochondrial respiratory chain inhibitors, hydrogen peroxide (H_2_O_2_) that does not directly induce mitochondrial superoxide generation (Lee et al., 2009), as indicated by MitoSOX Red (Figure S2e), failed to induce PINK1 accumulation or Parkin recruitment onto mitochondria, even in cells that were treated in combination with DNP (Figure S2f,g). These data suggest that mitochondrial oxidative stress has a role in PINK1 accumulation.

### 2.2 CHCHD4/GFER disulfide relay system is important for PINK1 accumulation

**Figure 2:**
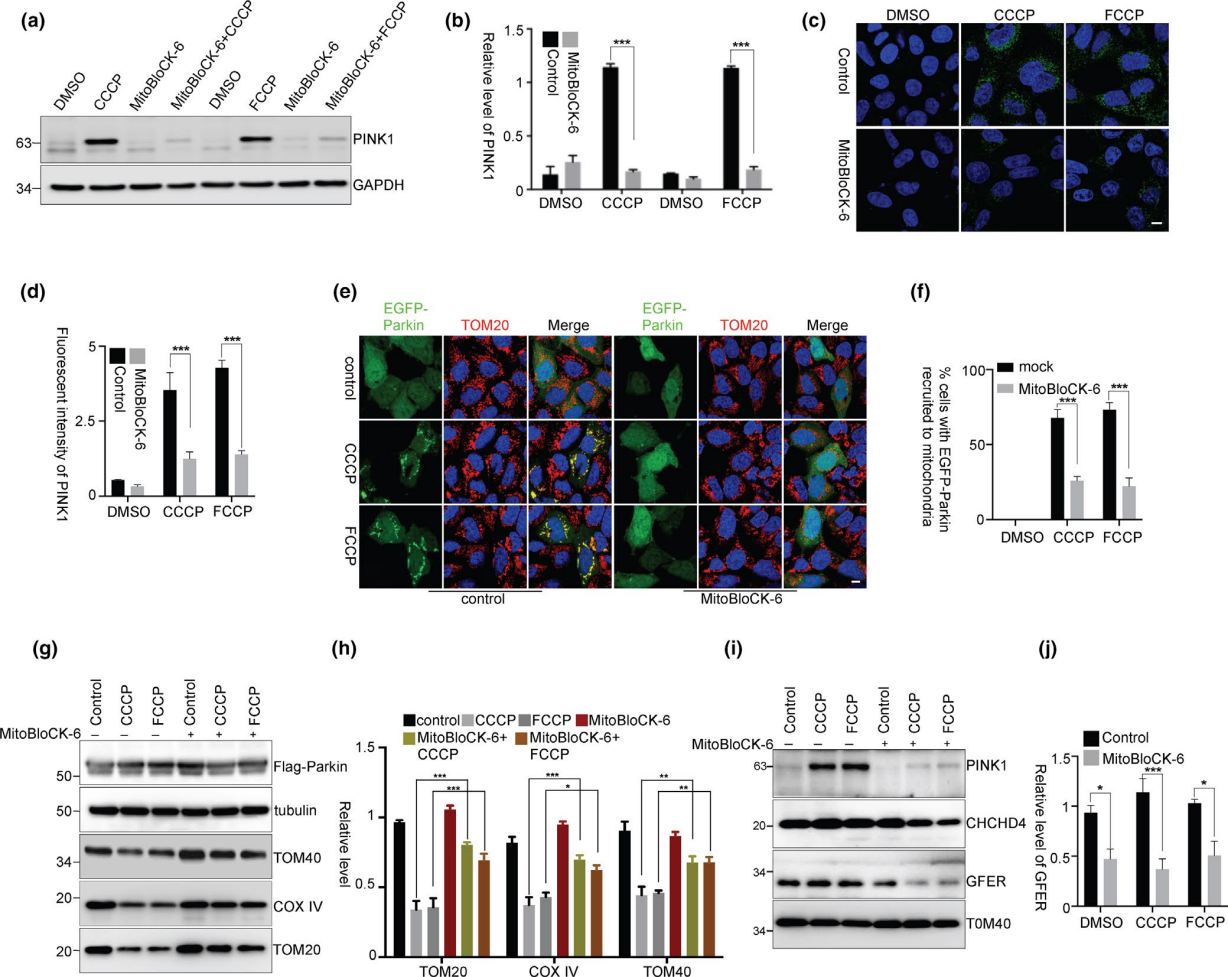
PINK1 accumulation requires mitochondrial disulfide relay system. (a, b) HEK293 cells were treated with MitoBloCK‐6 (50 μM) for 2 hr and then together with CCCP or FCCP for 3 hr. The relative levels of PINK1 to GAPDH from three independent experiments were shown in (b). Mean ± *SEM*, ***p < 0.001 by two‐way ANOVA. (c, d) HEK293 cells were treated as (a) and then subjected to immunocytochemical staining with PINK1 antibody (green). Fluorescent intensity of PINK1 was quantified, three replicates for each group and >10 images for each replicate (d). Mean ± *SEM*, ***p < 0.001 by one‐way ANOVA. Scale bar, 10 μm. (e, f) HEK293 cells were transfected with EGFP‐Parkin and then treated with MitoBloCK‐6 for 2 hr followed by CCCP or FCCP treatment for 3 hr. The cells were then subjected to immunocytochemical staining with TOM20 antibody (red). The percentage of cells with EGFP‐Parkin recruited to mitochondria was quantified (f), three replicates for each group, with >80 cells counted for each replicate. Mean ± *SEM*, ***p < 0.001 by two‐way ANOVA. Scale bar, 10 μm. (g, h) HEK293 cells were transfected with Flag‐Parkin and then treated with MitoBloCK‐6 for 2 hr followed by CCCP or FCCP treatment for 24 hr. The relative levels of TOM20, TOM40, and COX IV to tubulin from three independent experiments were shown in (h). Mean ± *SEM*, *p < 0.05, **p < 0.01, ***p < 0.001 by two‐way ANOVA. (i, j) HEK293 cells were pretreated with MitoBloCK‐6 and then treated with CCCP or FCCP for 3 hr. Mitochondria were isolated for immunoblotting. The relative levels of GFER to TOM40 from three independent experiments were quantified (j). Mean ± *SEM*, *p < 0.05, ***p < 0.001 by two‐way ANOVA

The CHCHD4/GFER disulfide relay system, a mammalian homologue of yeast Mia40/Erv1 redox system that transfers proteins into the IMS, is sensitive to mitochondrial oxidative status (Mesecke et al., 2005). We therefore investigated whether the oxidative status in mitochondria affects the IMS disulfide relay system to recognize PINK1 and to assist PINK1 accumulation. In HEK293 cells (Figure 2a,b) or SH‐SY5Y neuroblastoma cells (Figure S3a) that were pretreated with the mitochondrial CHCHD4/GFER system inhibitor MitoBloCK‐6, CCCP‐ or FCCP‐induced PINK1 accumulation was remarkably inhibited. And the CCCP‐ or FCCP‐induced PINK1 mitochondrial enrichment was also inhibited in cells that were pretreated with MitoBloCK‐6 (Figure 2c,d). Moreover, MitoBloCK‐6 also inhibited CCCP‐ or FCCP‐induced Parkin recruitment onto mitochondria (Figure 2e,f) and mitophagy (Figure 2g,h). However, MitoBloCK‐6 itself did not reverse CCCP‐ or FCCP‐induced ΔΨm loss (Figure S3b) or oxidative status in mitochondria (Figure S3c). In addition, with normal ΔΨm, MitoBloCK‐6 itself neither affected PINK1 transport to matrix for its cleavage nor influenced its release to the cytosol for the proteasomal degradation, as the processed PINK1 was accumulated after MG132 treatment (Figure S3d). Furthermore, the mitochondrial GFER that depends on CHCHD4/GFER system for IMS transport was greatly reduced in MitoBloCK‐6 treated cells (Figure 2i,j), suggesting that MitoBloCK‐6 inhibits the IMS CHCHD4/GFER system. Thus, these data indicate that the mitochondrial disulfide relay system has a role in PINK1 accumulation when ΔΨm is lost.

### 2.3 CHCHD4 interacts with PINK1 for OMM stabilization

**Figure 3:**
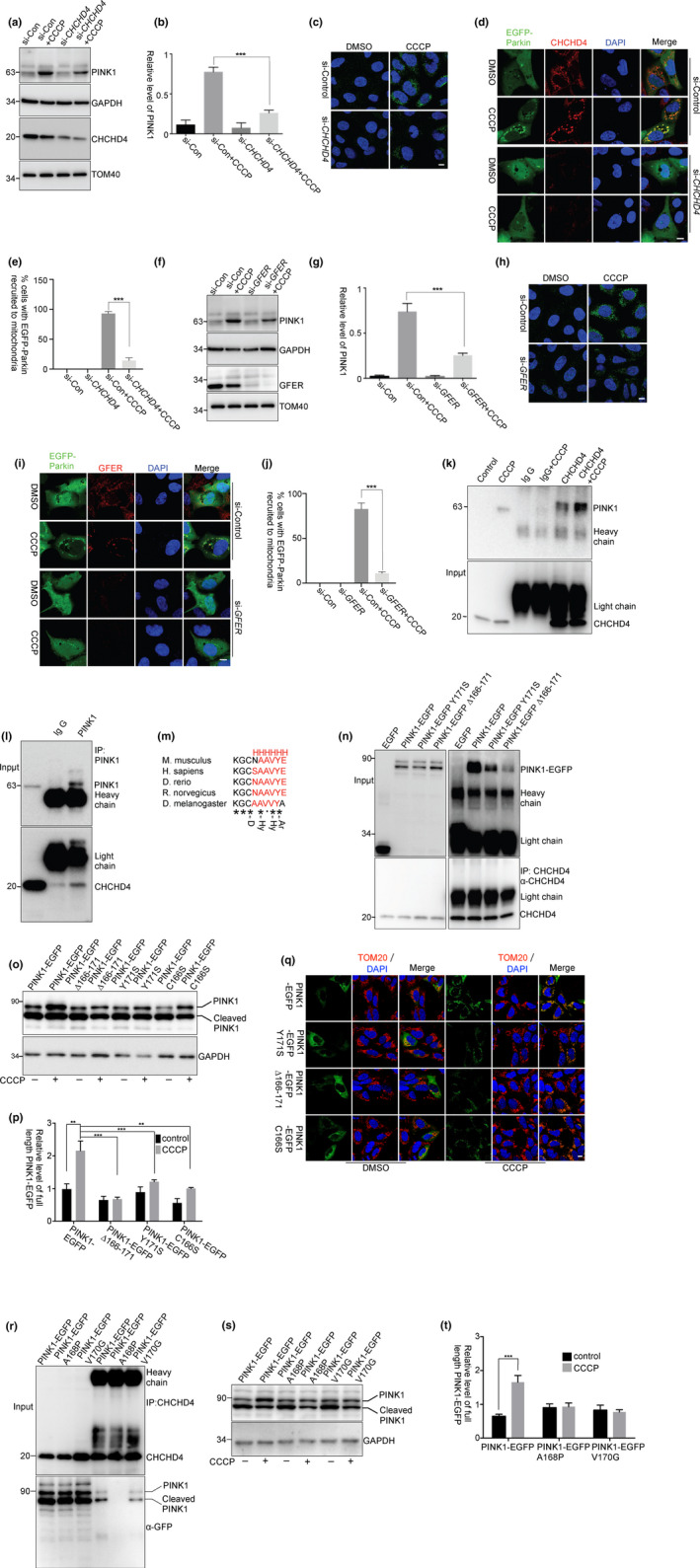
The activated CHCHD4 interacted with PINK1. (a–j) HEK293 cells were transfected with siRNAs against CHCHD4 (a–e) or GFER (f–j) for 72 hr. (a–c and f–h) The cells were then treated with CCCP for 3 hr. After treatment, the cells were subjected to immunoblotting or immunocytochemical staining with PINK1 antibody. (b, g) The relative levels of PINK1 to GAPDH from (a) and (f) with three independent experiments were quantified, respectively. (d, i) The cells were subjected to the same treatment as in a but the cells were transfected with EGFP‐Parkin after siRNAs transfection. (e, j) The percentage of cells with EGFP‐Parkin recruited to mitochondria was quantified from (d) and (i), respectively, three replicates for each group, with >80 cells counted for each replicate. Mean ± *SEM*, ***p < 0.001 by one‐way ANOVA. Scale bar, 10 μm. (k, l) HEK293 cells were treated with CCCP for 2 hr. The cells lysed and precipitated with anti‐CHCHD4 antibodies (k) or anti‐PINK1 antibody (l). (m) α‐helix prediction of PINK1 using PSIPRED (http://bioinf.cs.ucl.ac.uk/psipred/) shows that the α‐helix structure is conserved in different species. Consensus symbols: H, α‐helix; D, docking residue; Hy, hydrophobic residue; Ar, aromatic residue; “*”, identical residues; “:”, conserved substitution; and “,”, semi‐conserved substitution. (n) HEK293 cells were transfected with EGFP, PINK1‐EGFP, PINK1‐EGFP Y171S, or PINK1‐EGFP ∆166‐171 (AA166‐171 deletion) for 24 hr. The cells were lysed for immunoprecipitation with anti‐CHCHD4 antibody. The relative levels of PINK1‐EGFP, PINK1‐EGFP Y171S, and PINK1‐EGFP ∆166‐171 in the precipitants as compared to their inputs are 5.42, 3.31, and 1.01, respectively. (o–q) HEK293 cells were transfected with PINK1‐EGFP, PINK1‐EGFP Y171S, PINK1‐EGFP C166S, or PINK1‐EGFP ∆166‐171 for 24 hr, followed by treatments with CCCP for 3 hr. The relative levels of full‐length PINK1‐EGFP to GAPDH from three independent experiments were quantified (p). Mean ± *SEM*, **p < 0.01, ***p < 0.001 by two‐way ANOVA. Scale bar, 10 μm. (r) HEK293 cells were transfected with PINK1‐EGFP, PINK1‐EGFP A168P, and PINK1‐EGFP V170G for 24 hr. The cells were lysed for immunoprecipitation with anti‐CHCHD4 antibody. The relative levels of PINK1‐EGFP, PINK1‐EGFP A168P, and PINK1‐EGFP V170G in the precipitants as compared to their inputs are 0.73, 0.20, and 0.39, respectively. (s, t) HEK293 cells were transfected with PINK1‐EGFP, PINK1‐EGFP A168P, and PINK1‐EGFP V170G for 24 hr, followed by treatments with CCCP for 3 hr. The relative levels of full‐length PINK1‐EGFP to GAPDH from three independent experiments were quantified (t). Mean ± *SEM*, ***p < 0.001 by one‐way ANOVA

We used small interfering RNA to reduce the CHCHD4 or GFER expression to evaluate the effects of the CHCHD4/GFER system on PINK1 accumulation. In *CHCHD4* knockdown cells, CCCP‐induced PINK1 accumulation (Figure 3a–c) or Parkin translocation onto mitochondria (Figure 3d,e) was significantly decreased. Similar results were obtained in *GFER* knockdown cells (Figure 3f–j). Moreover, the processed PINK1 was accumulated in both control and *CHCHD4* knockdown cells that were treated with MG132 (Figure S3e), suggesting that *CHCHD4* knockdown does not influence PINK1 transporting into the IMM for cleavage and releasing to the cytosol for degradation. Thus, these data further indicate that the CHCHD4/GFER system assists PINK1 accumulation when ΔΨm is lost.

The CHCHD4/GFER system transfers proteins into the IMS by a direct interaction between CHCHD4 and substrate (Hangen et al., 2015; Mesecke et al., 2005). To identify whether CHCHD4 interacts with PINK1, an anti‐CHCHD4 antibody was used for immunoprecipitation assay (Figure S4). The interactions between PINK1 and CHCHD4 were observed in cells that were treated with CCCP (Figure 3k,l). For the IMS targeting, the substrate interaction with CHCHD4 requires an IMS‐targeting sequence (ITS) that is composed of an amphipathic helix with critical hydrophobic and aromatic residues on the side of the docking cysteine (Sideris et al., 2009). In PINK1, the Cys166 is conserved in different species and adjacent to an amphipathic helix (amino acids (AA) 166‐172) with hydrophobic and aromatic residues (Y171) (Figure 3m). Interestingly, the PINK1‐EGFP amphipathic helix deletion mutant (PINK1 ∆166‐171) or Y171S point mutant decreased its interaction with CHCHD4 (Figure 3n). Moreover, PINK1‐EGFP ∆166‐171, Y171S, or C166S mutant decreased accumulation on mitochondria in the cells that were treated with CCCP (Figure 3o–q). Most importantly, the PD‐associated mutants, PINK1 A168P and V170G, also decreased their interactions with CHCHD4 (Figure 3r) and their accumulation in the cells that were treated with CCCP (Figure 3s,t). Although the sequence in PINK1 seems not an ITS, it affects PINK1 interaction with CHCHD4 and accumulation on the OMM.

### 2.4 Mitochondrial oxidative stress increases CHCHD4 activity to promote PINK1 accumulation

**Figure 4:**
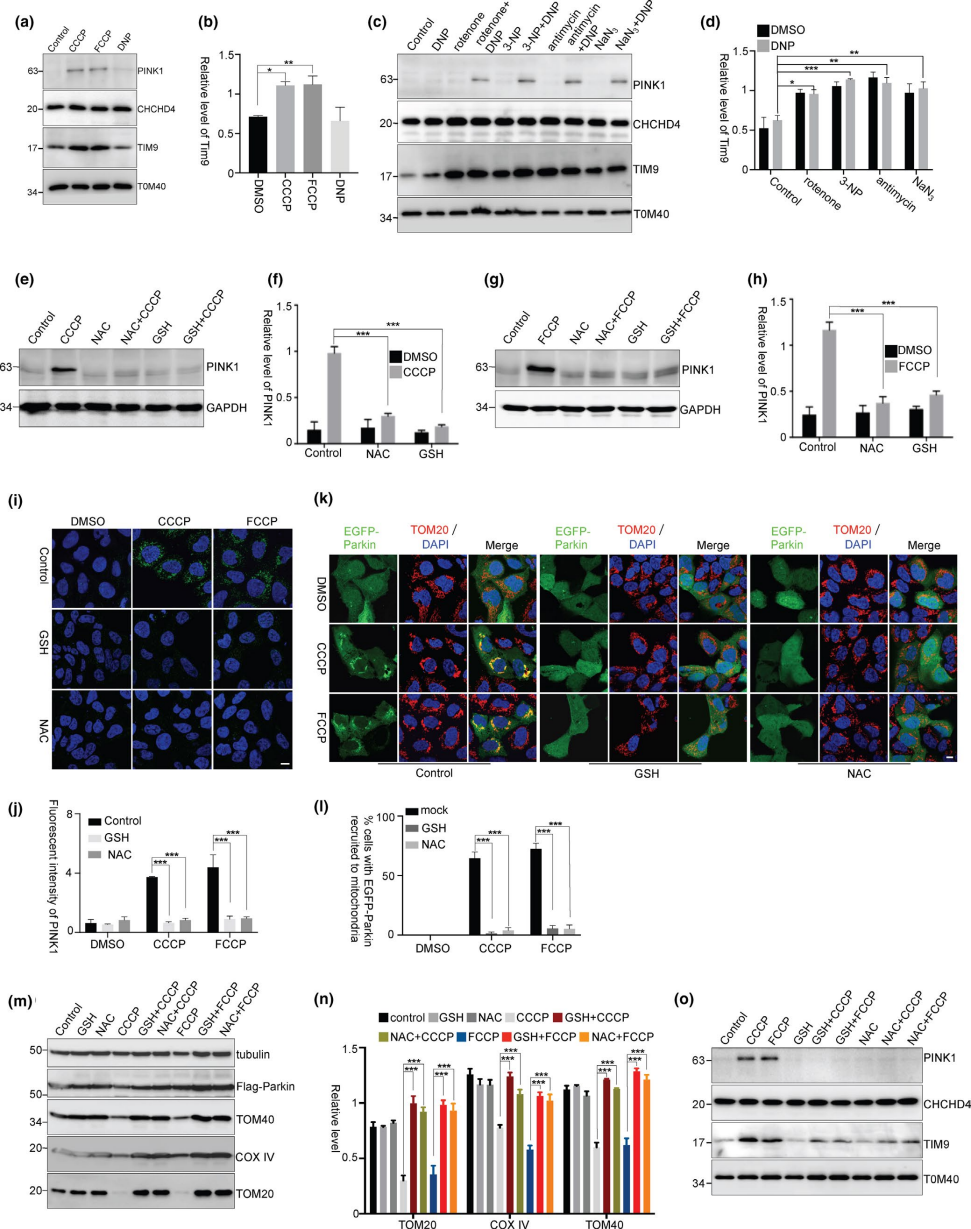
Mitochondrial oxidative stress is required for PINK1 accumulation. (a, b) HEK293 cells were pretreated with CCCP, FCCP, or DNP for 3 hr. Mitochondria were isolated for immunoblotting. The relative levels of TIM9 to TOM40 from three independent experiments were shown in (b). Mean ± *SEM*, *p < 0.05, **p < 0.01 by one‐way ANOVA. (c, d) HEK293 cells were pretreated with rotenone, 3‐NP, antimycin, or NaN_3_ and then treated with DNP for 3 hr. Mitochondria were isolated for immunoblotting. (d) The relative levels of TIM9 to TOM40 from three independent experiments were quantified. Mean ± *SEM*, *p < 0.05, **p < 0.01, ***p < 0.001 by two‐way ANOVA. (e–j) HEK293 cells were treated with GSH (10 mM) or NAC (10 mM) for 2 hr followed by CCCP (5 μM) (e,f) or FCCP (5 μM) (g, h) treatment for 3 hr. (f, h) The relative levels of PINK1 to GAPDH from (e) and (g) with three independent experiments were quantified, respectively. (j) Fluorescent intensity of PINK1 from (i) was quantified, three replicates for each group and >10 images for each replicate. Mean ± *SEM*, *** *p* < 0.001 by two‐way ANOVA. Scale bar, 10 μm. (k–n) HEK293 cells were transfected with EGFP‐Parkin (k and l) or Flag‐Parkin (m and n) and then treated with GSH or NAC for 2 hr followed by CCCP or FCCP treatment for 3 hr (k and l) or 24 hr (m and n). (l) The percentage of cells with EGFP‐Parkin recruited to mitochondria from k was quantified, three replicates for each group, with >80 cells counted for each replicate. (n) The relative levels of TOM20, TOM40, and COX IV to tubulin from m with three independent experiments were quantified. Mean ± *SEM*, *** *p* < 0.001 by two‐way ANOVA. Scale bar, 10 μm. (o) HEK293 cells were pretreated with GSH or NAC for 2 hr and then treated with CCCP or FCCP for 3 hr. Mitochondria were isolated for immunoblotting

The activity of CHCHD4/GFER system is important for the IMS protein import and folding, which depends on the oxidative status in the IMS. The PINK1 accumulation requires the mitochondrial oxidative stress, suggesting that the increased activity of CHCHD4/GFER system may promote the interactions between PINK1 and CHCHD4 for PINK1 OMM accumulation. In our observations, mitochondrial CHCHD4 levels were not affected in cells that were treated with CCCP, FCCP, or DNP for 3 hr (Figure 4a). However, mitochondrial TIM9, a substrate of CHCHD4/GFER system, was remarkably increased in CCCP‐ or FCCP‐treated cells, but not in DNP‐treated cells (Figure 4a,b). Consistently, mitochondrial TIM9 levels were substantially increased in cells that were pretreated with mitochondrial respiratory chain inhibitors (Figure 4c,d). Thus, the mitochondrial oxidative stress improves CHCHD4/GFER system activity and promotes the interactions between PINK1 and CHCHD4.

**Figure 5:**
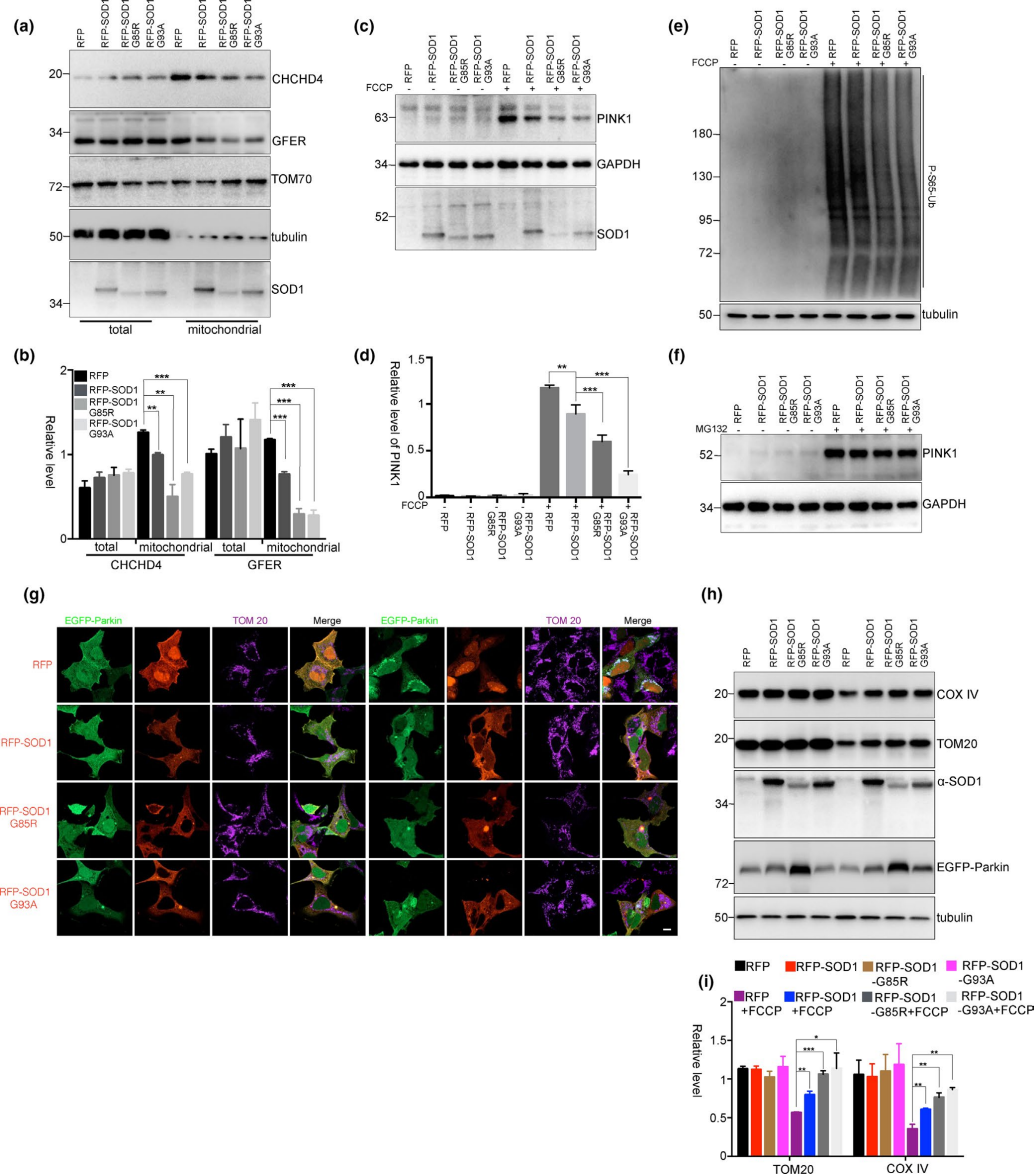
SOD1 mutants inhibit PINK1 accumulation and mitophagy. (a) and (b) HEK293 cells were transfected with RFP, RFP‐SOD1, RFP‐SOD1 G85R, or RFP‐SOD1 G93A for 48 hr. Then, the mitochondria were isolated for immunoblot analysis. The relative levels of CHCHD4 and GFER to TOM70 from three independent experiments were shown in (b). Mean ± *SEM*, **p < 0.01, ***p < 0.001 by one‐way ANOVA. (c–f) HEK293 cells were transfected with RFP, RFP‐SOD1, RFP‐SOD1 G85R, or RFP‐SOD1 G93A for 48 hr and treated with FCCP (5 mM) for 3 hr (c–e) or MG132 (10 mM) for 3 hr (f). The endogenous PINK1 (c and f) and phospho‐Ub (Ser65) (e) were detected using immunoblot analyses. The relative levels of PINK1 to GAPDH from three independent experiments were quantified (d). Mean ± *SEM*, **p < 0.01, ***p < 0.001 by one‐way ANOVA. (g–i) HEK293 cells were co‐transfected with RFP, RFP‐SOD1, RFP‐SOD1 G85R or RFP‐SOD1 G93A and EGFP‐Parkin, followed by treatments with FCCP (5 mM) for 3 hr (g) or FCCP (5 mM) for 24 hr (h) and (i). The relative levels of TOM20 and COX IV to tubulin from three independent experiments were shown in (i). Mean ± *SEM*, *p < 0.05, **p < 0.01, ***p < 0.001 by one‐way ANOVA. Scale bar, 10 μm

To identify the role of mitochondrial oxidative stress in PINK1 accumulation, we tested whether antioxidants could inhibit PINK1 accumulation. We observed that the antioxidant glutathione (GSH) or N‐acetyl‐cysteine (NAC) did not reverse ΔΨm loss (Figure S5a), but significantly reduced mitochondrial ROS levels in CCCP‐ or FCCP‐treated cells (Figure S5b and Figure 5c). Interestingly, CCCP‐ or FCCP‐induced PINK1 stabilization (Figure 4e–j) and Parkin recruitment onto mitochondria (Figure 4k,l) were inhibited by GSH or NAC. Moreover, mitochondrial degradation induced by CCCP was also inhibited by GSH or NAC (Figure 4m,n). However, the antioxidant itself did not influence PINK1 importing to the IMM for PINK1 processing (Figure S5d). In addition, antioxidants did not reverse oligomycin in combination with antimycin (OA) treatment‐induced ΔΨm loss, but inhibited OA‐induced PINK1 accumulation (Figure S5e–g). Moreover, in cells that were pretreated with antioxidants, CCCP‐ or FCCP‐mediated upregulation of CHCHD4/GFER activity was significantly inhibited as indicated by mitochondrial TIM9 levels (Figure 4o). These data further suggest that an oxidative status for CHCHD4/GFER system activity has a pivotal role in PINK1 accumulation.

To further confirm the dependence of PINK1 accumulation on CHCHD4 activity, we pretreated the cells with a low dose of rotenone to improve the activity of CHCHD4/GFER. CCCP‐ or FCCP‐induced PINK1 accumulation was significantly accelerated in rotenone‐pretreated cells (Figure S6a,b). A low dose of CCCP (2 μM) that effectively induced ΔΨm collapse but not increased mitochondrial ROS level (Figure S6c–e) failed to induce PINK1 accumulation or Parkin recruitment to mitochondria (Figure S6f–i). However, in cells that were pretreated with mitochondrial respiratory chain inhibitors, a low dose of CCCP induced PINK1 accumulation and Parkin recruitment to mitochondria (Figure S6j–m). Thus, these data suggest that loss of ΔΨm alone is not sufficient for PINK1 accumulation and that mitochondrial oxidation‐induced CHCHD4 activity is necessary for PINK1 accumulation on ΔΨm lost mitochondria.

### 2.5 Mitochondrial SOD1 and its mutants influence CHCHD4/GFER activity and inhibit PINK1 accumulation

A considerable proportion of superoxide dismutase 1 (SOD1) is imported into mitochondrial IMS by copper chaperone for SOD1 (CCS) (Field, Furukawa, O'Halloran, & Culotta, 2003). Importantly, the SOD1 pathogenic mutants G85R and G93A are enriched in the IMS, which may contribute to mitochondrial dysfunction in amyotrophic lateral sclerosis (ALS) (Liu et al., 2004; Mattiazzi et al., 2002; Son et al., 2007). Because the SOD1 mutants retain almost all dismutase enzyme activity, the enrichment of mutant SOD1 in the IMS dysregulates the redox state and may inhibit the CHCHD4/GFER disulfide relay system. To examine this hypothesis, we detected mitochondrial CHCHD4 or GFER using isolated mitochondria from cells that were transfected with RFP‐SOD1 or RFP‐SOD1 G85R or RFP‐SOD1 G93A. The mitochondrial CHCHD4 and GFER were decreased in RFP‐SOD1‐transfected cells, and more decreased in RFP‐SOD1 G85R‐ or RFP‐SOD1 G93A‐transfected cells (Figure 5a,b), although the mitochondrial ΔΨm and ROS levels were not significantly changed in those cells (Figure S7a,b). Moreover, PINK1 accumulation was decreased in RFP‐SOD1‐transfected cells and more significantly decreased in RFP‐SOD1 G85R‐ or RFP‐SOD1 G93A‐transfected cells (Figure 5c,d). The OMM PINK1 phosphorylates ubiquitin Ser65 (p‐S65‐Ub) to activate Parkin E3 ubiquitin ligase activity (Koyano et al., 2014). In RFP‐SOD1 G85R‐ or RFP‐SOD1 G93A‐transfected cells, FCCP‐induced p‐S65‐Ub was largely reduced, further suggesting an inhibition of PINK1 accumulation by SOD1 G85R and G93A (Figure 5e). Meanwhile, the processed PINK1 was not changed in SOD1‐ or mutant SOD1‐transfected cells (Figure 5f), indicating that SOD1 and its mutants do not influence the importing of PINK1 to mitochondria for process, but influence PINK1 accumulation on the OMM. In cells that were co‐transfected with EGFP‐Parkin and RFP‐SOD1 or mutant SOD1, the recruitment of EGFP‐Parkin onto mitochondria was decreased after FCCP treatment (Figure 5g). Meanwhile, the mitochondrial protein degradation was also inhibited (Figure 5h–i). Thus, the abnormal accumulation of SOD1 in the IMS suppresses Parkin‐mediated mitophagy, which may contribute to damaged mitochondrial accumulation in spinal motor neurons of ALS patients (Tan, Pasinelli, & Trotti, 2014).

## 3 DISCUSSION

It is well known that PINK1 accumulates on the OMM of damaged mitochondria to induce Parkin activation and mitophagy as a mechanism for selective mitochondrial elimination. In addition to the induction of PINK1 accumulation by an overexpression of misfolded proteins in mitochondrial matrix, the accumulation of PINK1 depends on loss of ΔΨm induced by the mitochondrial uncoupling drugs, including CCCP and FCCP (Jin & Youle, 2013). However, DNP or a lower dose of CCCP that is sufficient to collapse ΔΨm (Figure S1a) does not induce PINK1 accumulation or Parkin recruitment onto the OMM (Figure 1a–d). Thus, loss of ΔΨm is not sufficient for PINK1 accumulation. H_2_O_2_ at a concentration of 10 μM does not induce mitochondrial ROS generation (Lee et al., 2009). In our observations, rotenone but not H_2_O_2_ induces mitochondrial superoxide generation, indicated by the staining of MitoSOX Red, a mitochondrial superoxide indicator that is selectively targeted to mitochondria and oxidized by superoxide in living cells (Figure S2e). In cells pretreated with rotenone or other mitochondrial respiratory chain inhibitors, but not with H_2_O_2_, DNP or a lower dose of CCCP restores its ability to induce PINK1 accumulation on the OMM, suggesting that the formation of superoxide in mitochondria is critical for PINK1 accumulation. The mitochondrial matrix‐targeted PINK1 transport is dependent on mitochondrial membrane potential (Jin et al., 2010). Loss of mitochondrial membrane potential inhibits PINK1 processing, leading to PINK1 accumulation on the OMM. Besides loss of mitochondrial membrane potential, mitochondrial oxidative stress promotes PINK1 accumulation and the recruitment of Parkin onto mitochondria (Xiao, Deng, et al., 2017). Moreover, the mitochondrial oxidative stress also promotes mitochondrial degradation in a PINK1‐independent pathway (Dagda et al., 2009; Xiao, Goh, et al., 2017), further suggesting that loss of mitochondrial membrane potential is a prerequisite for mitophagy (Jin & Youle, 2013), but not the only mechanism for mitophagy induction.

It has been reported that an acidification of the cytosol was suggested to induce the mitophagy through PINK1/Parkin‐dependent and independent pathway after FCCP/CCCP treatment (Berezhnov et al., 2016). The cytosolic acidification‐induced mitophagy by FCCP is PINK1/parkin‐independent in short time (2 hr), but a prolonged acidification (24‐hr incubation with FCCP) induces Parkin‐dependent mitophagy. The redistribution of the H^+^ within mitochondria, lysosome, and cytosol by FCCP may contribute to the early initiation of mitophagy, which is independent of PINK1/Parkin pathway. It seems that the acidification of cytosol‐induced mitophagy differs from membrane potential loss‐induced PINK1‐mediated mitophagy.

The most important role of mitochondrial oxidative stress in PINK1 accumulation is that the mitochondrial oxidative status is tightly associated with the activity of the redox system. Import of the IMS proteins depends on the CHCHD4‐GFER machinery, a redox system that transfers disulfide bonds to its substrates (Chacinska et al., 2004; Sideris et al., 2009). The redox cycle of Mia40/CHCHD4 and Erv1/GFER are important for the IMS protein import and folding, which depends on the oxidative status in the IMS. In our observation, the mitochondrial CHCHD4 and GFER do not significantly decreased with a 2‐hr treatment of NAC or GSH. However, the CHCHD4/GFER system substrate, the mitochondrial small TIM family protein TIM9 (Chacinska et al., 2004), is remarkably decreased in NAC‐ or GSH‐treated cells, suggesting an inhibition of the activity but not the protein levels of CHCHD4/GFER system by anti‐oxidants. Both knockdown of *CHCHD4* or *GFER* by siRNA and inhibition of CHCHD4/GFER oxidase activity by chemicals decrease CCCP‐induced PINK1 accumulation. In addition, antioxidants abolish CCCP‐induced PINK1 accumulation. As the reduced condition in mitochondria counteracts the formation of disulfide bonds, the redox system activated by mitochondrial oxidative stress is important for activating CHCHD4. It is also possible that the activated redox system may induce a formation of disulfide bonds in PINK1 through the interactions between CHCHD4 and PINK1, which reshuffles the disulfide bonds to stabilize PINK1,

PINK1 has an N‐terminal matrix targeting sequence and a transmembrane domain (Lin & Kang, 2010), and targets dually to the IMM and the OMM (Zhou et al., 2008). After PINK1 cleaved by PARL, the processed PINK1 is released into the IMS or the cytosol (Deas et al., 2011; Jin et al., 2010; Meissner, Lorenz, Weihofen, Selkoe, & Lemberg, 2011). It has been reported that PINK1 binds to the TOM complex, which promotes PINK1 stabilization (Lazarou et al., 2012) and lateral release (Hasson et al., 2013) on the OMM for accumulation. With an oxidative status in the IMS, CHCHD4 increases its interaction with PINK1, which avoids PINK1 releasing to the cytosol and increases its retention on the OMM. The short stay of PINK1 with the TOM complex may promote its recruiting other proteins, like TOM7, to assist PINK1 lateral transport for its accumulation on the OMM (Sekine et al., 2019).

As CHCHD4/GFER disulfide relay system also transports itself into the IMS, decreases of CHCHD4 or GFER by overexpression of SOD1 or its G85R or G93A mutant suggest that SOD1 or its G85R or G93A mutant in the IMS can dysregulate the redox state and inhibit CHCHD4/GFER system. Consistent with the effects of anti‐oxidants on inhibiting PINK1 mitochondrial accumulation, SOD1 or its G85R or G93A mutants also interfere with PINK1 accumulation or mitochondrial degradation induced by FCCP. Thus, the present study suggests that the mitochondrial SOD1 mutants not only induce mitochondrial toxicity, but also inhibit the mitophagy process, which may contribute to mitochondrial pathology observed in ALS patients or SOD1 mutants transgenic mouse models (Tan et al., 2014; Xie et al., 2015).

In summary, we reveal a molecular mechanism underlying PINK1 accumulation, showing that loss of ΔΨm with an activation of the redox system contributes to PINK1 accumulation. An inhibition of the redox system by antioxidants or SOD1 mutants interferes with CHCHD4/GFER disulfide relay system and disrupts PINK1 accumulation and mitophagy. Thus, the redox system plays roles in the clearance of damaged mitochondria, which may be a potential therapeutic target for mitochondrial dysfunction‐related neurodegenerative diseases.

## 4 EXPERIMENTAL PROCEDURES

### 4.1 Cell culture and transfection

HEK293 cells were cultured in Dulbecco’s modified Eagle’s medium (Thermo Scientific) supplemented with 10% fetal calf serum (Thermo Scientific), including penicillin (100 U/ml) and streptomycin (100 μg/ml) (Thermo Scientific). The cells were transfected with the indicated plasmids with Lipofectamine 3000 reagent (Thermo Scientific) according to the manufacturer’s instructions. For the RNA interference experiments, the cells were transfected with oligonucleotides against target RNA with Lipofectamine RNAiMax (Thermo Scientific). The siRNAs targeting *CHCHD4* (GS131474) and *GFER* (GS2671) were purchased from Qiagen.

### 4.2 Cell treatment

The reagents, FCCP, CCCP, DNP, 3‐NP, antimycin, oligomycin, rotenone, NaN_3_, GSH, and NAC, were purchased from Sigma‐Aldrich. For mitochondrial depolarization, cells were treated with CCCP (5 μM), FCCP (5 μM), DNP (0.5 mM), or oligomycin (10 μM) together with antimycin (4 μM) for 3 hr. For inducing mitochondrial oxidative stress, cells were treated with rotenone (1 μM), 3‐NP (10 mM), antimycin (10 μM), NaN_3_ (5 mM), and H_2_O_2_ (10 μM) for 2 hr. For inhibiting mitochondrial oxidative stress, cells were treated with antioxidant GSH (10 mM) or NAC (10 mM) for 2 hr. For inhibiting the IMS disulfide relay system activity, cells were treated with MitoBloCK‐6 (Millipore) (50 μM) for 2 hr. For inhibiting the proteasomal activity, cells were treated with MG132 (10 μM) (Millipore). For the measurement of mitochondrial ROS levels, the cells were incubated in the serum‐free medium with fluorimetric probes, dichloro‐dihydro‐fluorescein diacetate (DCFH‐DA, 10 μM) (Sigma‐Aldrich) at 37°C for 20 min or MitoSOX Red (10 μM) (Thermo Scientific) at 37°C for 15 min. Next, the cells were washed three times with serum‐free medium. For DCFH‐DA detection, samples were analyzed at an excitation wavelength of 488 nm and an emission wavelength of 522 nm using a microplate reader (Synergy H1, BioTek). For measurement of the mitochondrial membrane potential, tetramethylrhodamine ethyl ester perchlorate (TMRE, 50 nM) (Sigma‐Aldrich) was used. The fluorescence intensity of TMRE (Ex/Em = 549/575 nm) was detected using a microplate reader (Synergy H1, BioTek).

### 4.3 Immunoprecipitation, immunoblotting, and immunofluorescence

Immunoprecipitation and immunoblotting analyses were carried out as described previously (Gao et al., 2015). Briefly, the cells were treated with CCCP (5 μM) for 2 hr and then lysed in buffer containing 50 mM Tris‐HCl (pH 7.5), 150 mM sodium chloride, 1 mM EDTA, and 1% Nonidet P‐40 supplemented with the protease inhibitor cocktail (Roche). The polyclonal anti‐CHCHD4 antibodies (Proteintech) and anti‐PINK1 antibody (CST, D8G3) were used to precipitate CHCHD4 protein. Mitochondrial isolation was performed using the mitochondrial isolation kit according to the manufacturer’s instructions (Abcam). The purified mitochondria were then lysed in SDS sample buffer for immunoblotting. The following antibodies were used for immunoblotting: polyclonal anti‐CHCHD4 antibodies, polyclonal anti‐GFER antibodies, polyclonal anti‐TOM40 antibodies, polyclonal anti‐COX IV antibodies, polyclonal anti‐TIM9 antibodies, and monoclonal anti‐tubulin antibody (Proteintech); polyclonal anti‐HA antibodies, monoclonal anti‐HA antibody, monoclonal anti‐GFP antibody, and monoclonal anti‐TOM20 antibody (Santa Cruz); polyclonal anti‐PINK1 antibodies (Novus Biology); monoclonal anti‐GAPDH antibody (Millipore); and monoclonal anti‐Flag antibody (Sigma‐Aldrich). The HRP‐conjugated anti‐mouse, anti‐rabbit, or anti‐rabbit light chain‐specific antibodies were purchased from Jackson ImmunoResearch. The following antibodies were used for immunofluorescence assays: rabbit monoclonal anti‐PINK1 antibody (CST, D8G3), polyclonal anti‐CHCHD4 antibodies (Proteintech), polyclonal anti‐GFER antibodies (Proteintech), and monoclonal anti‐TOM20 antibody (Santa Cruz). The secondary antibodies for immunocytochemistry were Alexa Fluor 568‐ or Alexa Fluor 488‐labeled anti‐rabbit or anti‐mouse antibodies (Life Technologies). The cells were observed using an inverted IX73 microscope (Olympus) or LSM710 confocal microscope (Zeiss).

### 4.4 Plasmids construction

EGFP‐Parkin, Flag‐Parkin, RFP‐SOD1, RFP‐SOD1 G85R, RFP‐SOD1 G93A, or HA‐PINK1 was described previously (Gao et al., 2015; Wang, Ying, & Wang, 2012; Wang, Guo, et al., 2014). The HA‐PINK1 mutants were constructed using the following primers: 5′‐aactatccctgtaccctgc‐3′/5′‐ccggccatggcccaggcctt‐3′ for HA‐PINK1‐D, 5′‐gccgcgggcccttgcgg‐3′/5′‐gccccaggcccgcaccac‐3′ for HA‐PINK1 C92A, 5′‐gccggccgggcagtctttctg‐3′/5′‐agggcccgcgcagcc‐3′ for HA‐PINK1 C96A, 5′‐gctcaggagatccaggcaa‐3′/5′‐ggccgagaccgccc‐3′ for HA‐PINK1 C125A, and 5′‐ggtcaggagatccaggca‐3′/5′‐ggccgagaccgccc‐3′ for HA‐PINK1 C125G. PINK1‐EGFP was created by subcloning PINK1 cDNA excised from HA‐PINK1 and inserted into pEGFP‐N3 vector. The PINK1‐EGFP mutants were constructed using the following primers: 5′‐tgtgtctgaagccaccatgc‐3′/5′‐gcagcactgcagcccttacc‐3′ for PINK1‐EGFP Y171S, 5′‐cagcagtgctgctgtgtatgaa‐3′/5′‐cccttaccaatggactgcccta‐3′ for EGFP ‐PINK1 C166S, 5′‐gaagccaccatgcctacatt‐3′/5′‐agcactgcagcccttaccaa‐3′ for PINK1‐EGFP ∆166‐171, 5′‐cctgctgtgtatgaagcc‐3′/5′‐actgcagcccttaccaat‐3′ for PINK1‐EGFP A168P, and 5′‐cctgctgtgtatgaagcc‐3′/5′‐cagcagcactgcagccct‐3′ for PINK1‐EGFP V170G.

### 4.5 Statistical analysis

Statistical data were analyzed and graphed using GraphPad Prism 6. Statistical comparison between groups and treatments was performed using one‐way analysis of variance (ANOVA) or two‐way ANOVA, followed by Tukey’s post‐test. A *p*‐value <0.05 was considered statistically significant. Data are presented as the mean ± *SEM*.

## CONFLICT OF INTEREST

All authors declare that they have no competing financial interest.

## AUTHOR CONTRIBUTIONS

F.G. and G.W. initiated this project and designed the experiments. F.G. performed most of the experiments. Y.Z repeated parts of experiments using CCCP, FCCP, anti‐oxidants, and mitochondrial respiratory chain inhibitors. X.H., Z.T., and H.R. performed parts of experiments on Parkin recruitment onto mitochondria using immunocytochemical staining. F.G. and G.W. wrote the manuscript, and all authors edited the manuscript.

## DATA AVAILABILITY STATEMENT

The data that support the findings of this study are available with following linkage: https://data.mendeley.com/datasets/2tfs93v673/draft?a=d6ebbc00‐b6cd‐4f75‐9652‐795ec7b47f97.

## Supporting information

Figures S1‐S7
