# Supplementary material for: Dependence of PINK1 accumulation on mitochondrial redox system
Source: Aging Cell. 2020 Aug 11;19(9):e13211. doi: 10.1111/acel.13211 (PMC7511888; doi:10.1111/acel.13211)
Supplement: Supplementary file 1 — Figures S1‐S7 [file ACEL-19-e13211-s001.pdf]

Supplementary Figures

Dependence of PINK1 accumulation on mitochondrial redox system and transport machineries

Feng Gao[1,2*] , Yan Zhang[1], Xiaoou Hou[1], Zhouteng Tao[1,3], Haigang Ren[1], Guanghui Wang[1*]

[1]Laboratory of Molecular Neuropathology, Jiangsu Key laboratory of Neuropsychiatric Disorders & Department of Pharmacology, College of Pharmaceutical Sciences, Soochow University, Suzhou, Jiangsu 215123, China.
[2]The Neurodegenerative Disorder Research Center and Brain Bank, the CAS Key Laboratory of Brain Function and Diseases and School of Life Sciences and Medical Center, University of Science and Technology of China, Hefei, Anhui 230027, China.
[3]Center for Drug Safety Evaluation and Research, State Key Laboratory of New Drug Research, Shanghai Institute of Materia Medica, Chinese Academy of Sciences, 501 Haike Road, Shanghai 201203, China.

Figure S1

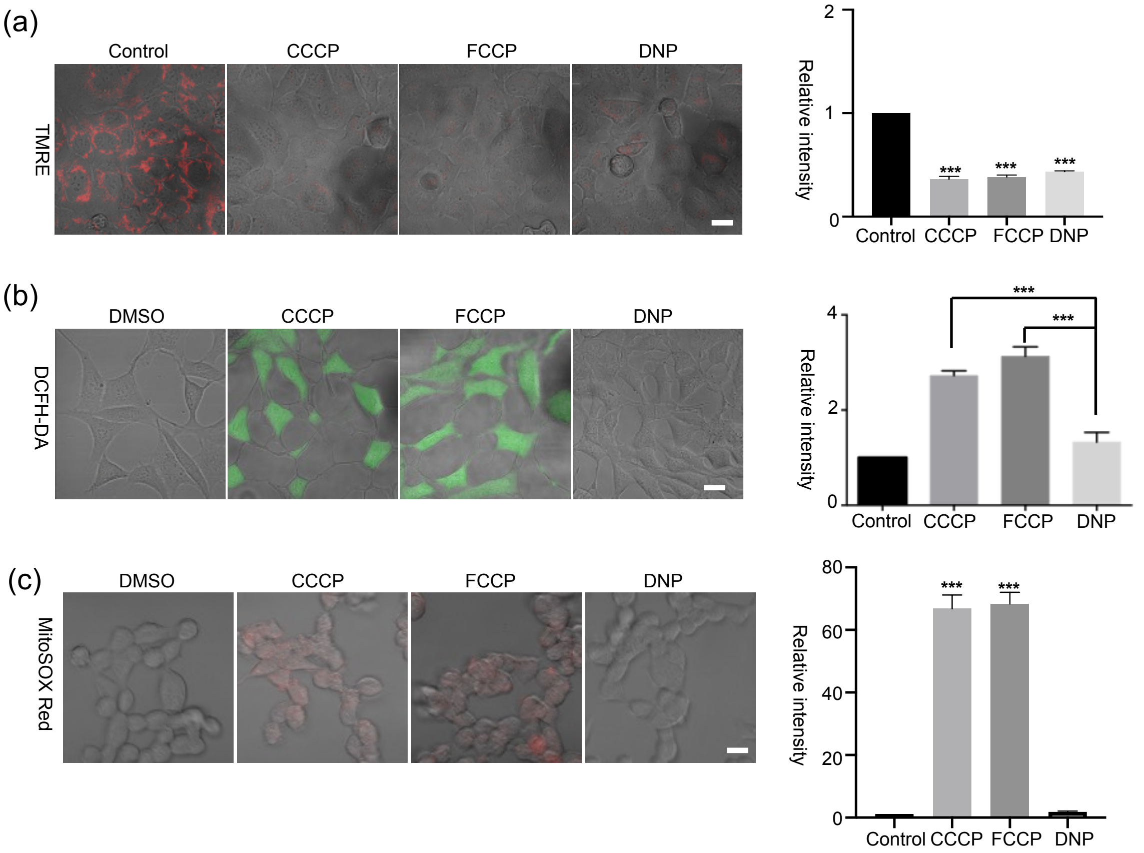

Figure S1. DNP induced mitochondrial ΔΨm loss, but did not mitochondrial ROS generation.
(a) 0.5 × 10^6 HEK293 cells are seeded per well and were treated with CCCP (5 μM), FCCP (5 μM) or DNP (0.5 mM) for 1 h. The cells were then stained with TMRE (a probe for detecting ΔΨm, 50 nM) for 15 min. All mitochondrial uncouplers induced mitochondrial ΔΨm loss. The relative fluorescence intensity compared with control was quantified, 3 replicates for each group. Mean ± SEM, *** $P < 0.001$ by one-way ANOVA. Scale bar, 20 μm.
(b) 0.5 × 10^6 HEK293 cells are seeded per well and were treated with CCCP (5 μM), FCCP (5 μM) or DNP (0.5 mM) for 2 h. The cells were then stained with DCFH-DA (a probe for detecting ROS generation, 10 μM) for 20 min. CCCP and FCCP, but not DNP, induced mitochondrial ROS generation as compared with control. The relative fluorescence intensity compared with control was quantified, 3 replicates for each group. Mean ± SEM, *** $P < 0.001$ by one-way ANOVA. Scale bar, 20 μm.
(c) HEK293 cells were treated with CCCP (5 μM), FCCP (5 μM) or DNP (0.5 mM) for 2 h and then stained with MitoSOX Red (a mitochondrial superoxide indicator). The relative fluorescence intensity compared with control was quantified, 3 replicates for each group. Mean ± SEM, *** $P < 0.001$ by one-way ANOVA. Scale bar, 20 μm.

Figure S2

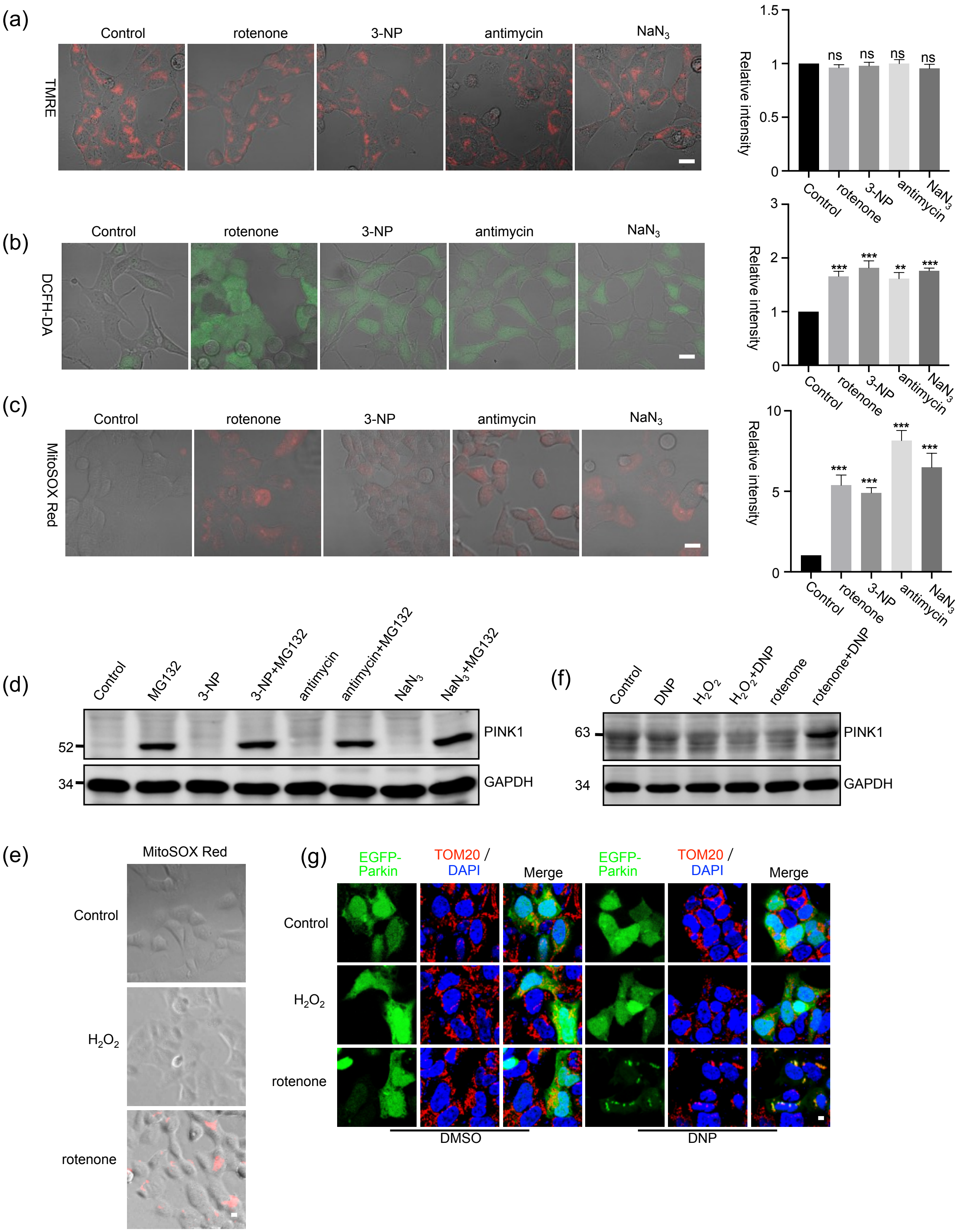

(a)
Control
rotenone
3-NP
antimycin
NaN3
TMRE
Relative intensity
1.5
1
0.5
0
ns
ns
ns
ns
Control
rotenone
3-NP
antimycin
NaN3
(b)
DCFH-DA
3
2
***
***
**
***
(c)
MitoSOX Red
10
5
(d)
MG132
3-NP+MG132
antimycin+MG132
NaN3+MG132
PINK1
GAPDH
52
34
(f)
DNP
H2O2
H2O2+DNP
rotenone+DNP
63
(e)
H2O2
(g)
EGFP-Parkin
TOM20 / DAPI
Merge
DMSO

Figure S2. H_2O_2 failed to induce mitophagy in combination with DNP treatment.

(a and b) 0.5 × 10^6 HEK293 cells are seeded per well and were treated with mitochondrial respiratory chain complex inhibitor 3-NP (5 mM), antimycin (10 μM), or NaN_3 (5 mM) for 2 h. The cells were then stained with TMRE (a) or DCFH-DA (b). No significant ΔΨm loss was observed in the cells that were treated with mitochondrial respiratory chain complex inhibitors (a). And all mitochondrial respiratory chain complex inhibitors induced mitochondrial ROS generation (b). Quantitative data are shown as relative fluorescence intensity compared with control, 3 replicates for each group. Mean ± SEM, ns: not significant, ** $P < 0.001$, *** $P < 0.001$ by one-way ANOVA. Scale bar, 20 μm.

(c) HEK293 cells were treated with mitochondrial respiratory chain complex inhibitor 3-NP (5 mM), antimycin (10 μM), or NaN_3 (5 mM) for 2 h and then stained with MitoSOX Red. Quantitative data are shown as relative fluorescence intensity compared with control, 3 replicates for each group. Mean ± SEM, *** $P < 0.001$ by one-way ANOVA. Scale bar, 20 μm.

(d) HEK293 cells were treated with 3-NP (5 mM), antimycin (10 μM) or NaN_3 (5 mM) together with MG132 (10 μM) or not for 4 h. After treatment, the cells were lysed for immunoblotting. MG132 or mitochondrial respiratory chain complex inhibitors did not affect PINK1 transporting into the IMM for processing.

(e) HEK293 cells were treated with H_2O_2 (10 μM) or rotenone (1 μM) for 2 h and then stained with MitoSOX Red. Rotenone but not H_2O_2 induced mitochondrial superoxide generation.

(f) HEK293 cells were pretreated with H_2O_2 (10 μM) or rotenone (1 μM) for 2 h and then treated with DNP (0.5 mM) or not for another 2 h. After treatment, the cells were lysed for immunoblotting. PINK1 accumulation was observed only in the cells that were treated with DNP in combination with rotenone.

(g) HEK293 cells were transfected with EGFP-Parkin. Twenty-four hour later, the cells were treated with H_2O_2 (10 μM) or rotenone (1 μM) for 2 h, followed by DNP treatment (0.5 mM) or not for another 2 h. EGFP-Parkin was recruited onto mitochondria only in the cells that were treated with DNP in combination with rotenone. Scale bar, 20 μm.

Figure S3

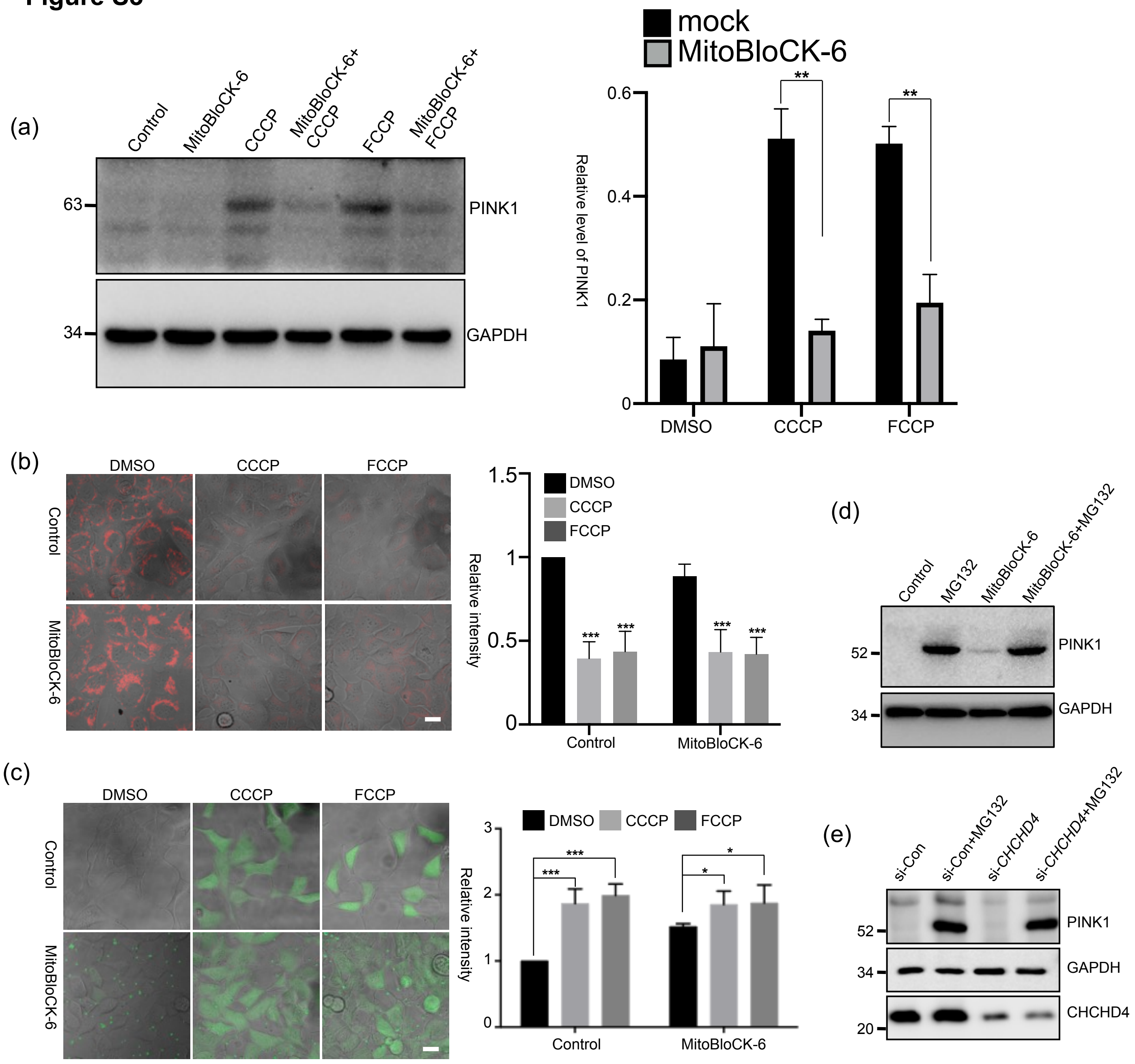

Figure S3. MitoBloCK-6 did not affect CCCP- or FCCP-induced ΔΨm loss and oxidative status in mitochondria.

(a) SH-SY5Y cells were were pretreated with MitoBloCK-6 (50 μM) for 2 h and then treated with CCCP (5 μM), FCCP (5 μM) or not for 3 h.The relative level of PINK1 to GAPDH from three independent experiments was quantified. Mean ± SEM, ** *P* < 0.01 by two-way ANOVA.

(b and c) HEK293 cells were pretreated with MitoBloCK-6 (50 μM) for 2 h and then treated with CCCP (5 μM), FCCP (5 μM) or not for 3 h. After treatment, the cells were stained with TMRE (b) or DCFH-DA (c). Without CCCP or FCCP, MitoBloCK-6 did not induce mitochondrial ΔΨm loss (b), but slightly increased mitochondrial ROS generation (c). Quantitative data are shown as relative fluorescence intensity compared with control, 3 replicates for each group. Mean ± SEM, * *P* < 0.05, *** *P* < 0.001 by two-way ANOVA. Scale bar, 20 μm.

(d) HEK293 cells were treated with MitoBloCK-6 (50 μM) along with MG132 (10 μM) or not for 4 h. The processed PINK1 was still observed in the cells that were treated with MitoBloCK-6 (50 μM) along with MG132.

(e) HEK293 cells were transfected with the negative control (si-Con) or siRNAs against *CHCHD4* for 72 h and the cells were then treated with MG132 (10 μM) or not for 3 h. The processed PINK1 was still observed in the cells in which *CHCHD4* was knocked down.

Figure S4

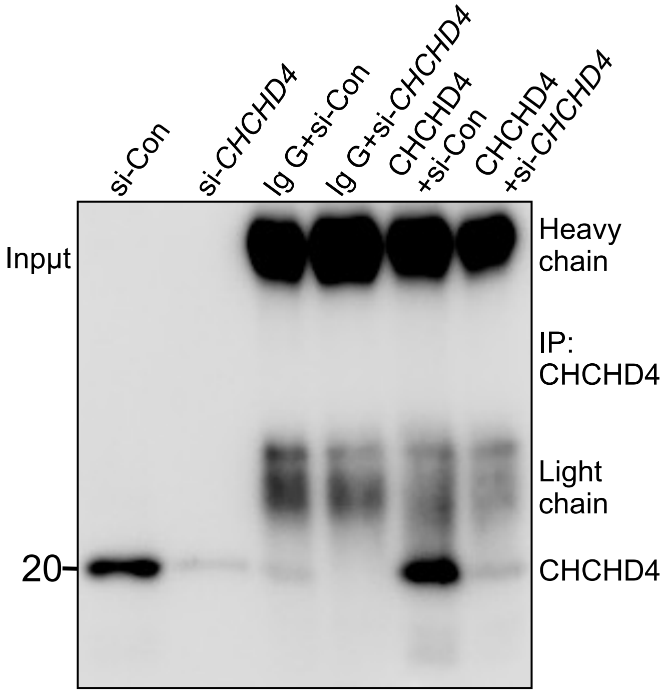

Figure S4. CHCHD4 antibody specificity testing.
HEK293 cells were transfected with the negative control (si-Con) or siRNAs against *CHCHD4* for 72 h and the cells were lysed for immunoprecipitation with anti-CHCHD4 antibody. The CHCHD4 was not precipitated from cells in which *CHCHD4* was knocked down, suggesting that the CHCHD4 antibody used in these experiments was specific.

Figure S5

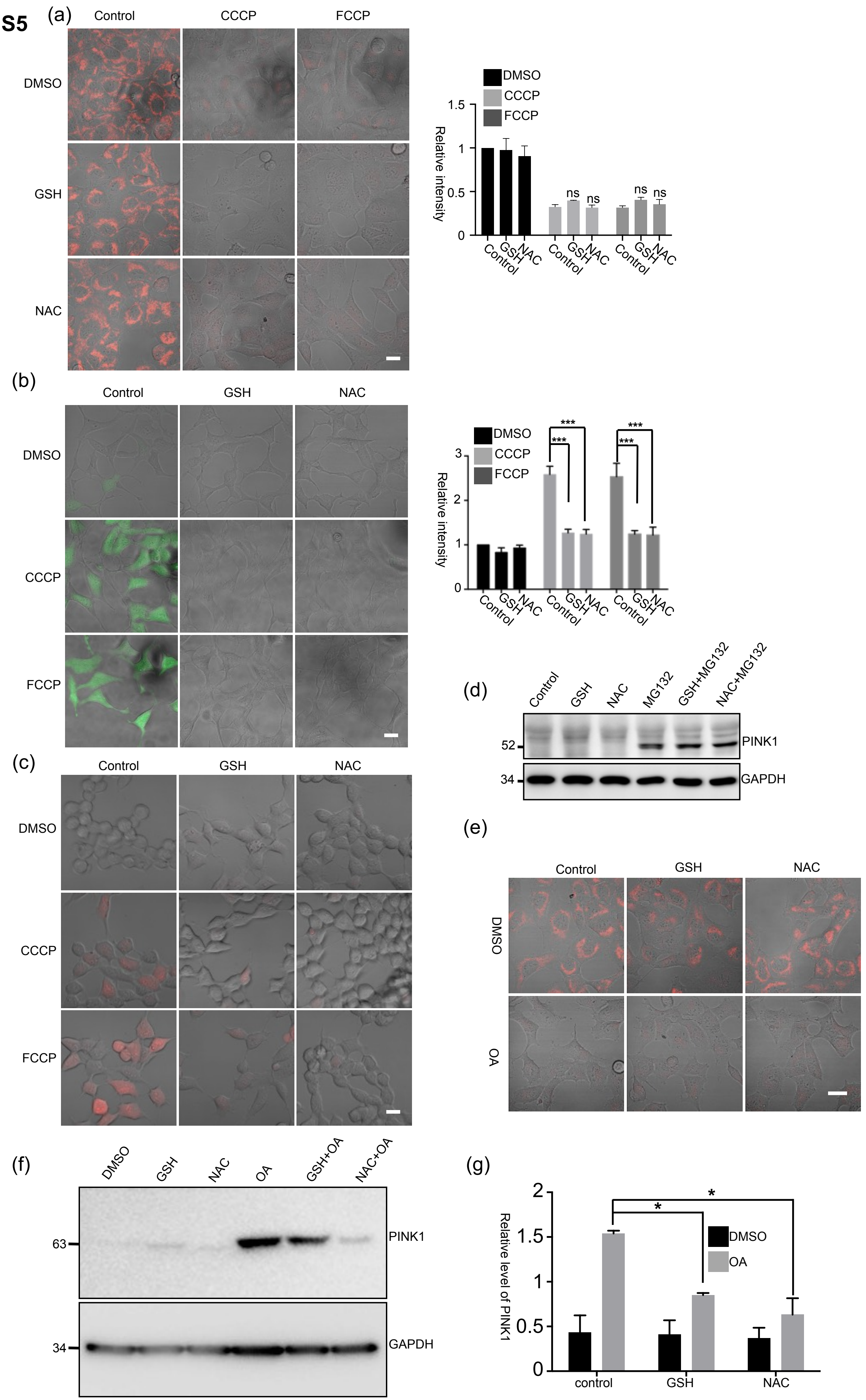

(a)
Control
CCCP
FCCP
DMSO
GSH
NAC
Relative intensity
ns
(b)
***
(c)
(d)
MG132
GSH+MG132
NAC+MG132
52
34
PINK1
GAPDH
(e)
OA
(f)
GSH+OA
NAC+OA
63
(g)
Relative level of PINK1
*
control

Figure S5. Anti-oxidants inhibited PINK1 accumulation.
(a and b) HEK293 cells were pretreated with GSH (10 mM) or NAC (10 mM) for 2 h and then treated with CCCP (5 μM), FCCP (5 μM) or not for 3 h. The cells were stained with TMRE (a) or DCFH-DA (b). NAC and GSH did not affect ΔΨm, but CCCP and FCCP induced ΔΨm loss no matter with or without antioxidants (a). CCCP or FCCP treatment increased mitochondrial ROS level, which was completely blocked by antioxidants (b). Quantitative data are shown as relative fluorescence intensity compared with control, 3 replicates for each group. Mean ± SEM, ns: not significant, *** $P < 0.001$ by one-way ANOVA. Scale bar, 20 μm.
(c) HEK293 cells were pretreated with GSH (10 mM) or NAC (10 mM) for 2 h and then treated with CCCP (5 μM), FCCP (5 μM) or not for 3 h and then stained with MitoSOX Red. Scale bar, 20 μm.
(d) HEK293 cells were treated with GSH (10 mM) or NAC (10 mM) in combination with MG132 (10 μM) or not for 4 h. MG132 inhibited PINK1 degradation but did not affect PINK1 transporting into the IMM for processing no matter with or without antioxidants.
(e) HEK293 cells were pretreated with GSH (10 mM) or NAC (10 mM) for 2 h and then treated with oligomycin (10 μM) and antimycin (4 μM) (OA) or not for 3 h. The cells were stained with TMRE. NAC and GSH did not affect ΔΨm, but OA induced ΔΨm loss no matter with or without antioxidants. Scale bar, 20 μm.
(f and g) HEK293 cells were treated with GSH (10 mM) or NAC (10 mM) in combination with OA or not for 3 h. The OA-induced PINK1 accumulation was inhibited in antioxidants-pretreated cells. The relative level of PINK1 to GAPDH from three independent experiments was quantified. Mean ± SEM, * $P < 0.05$ by one-way ANOVA.

Figure S6

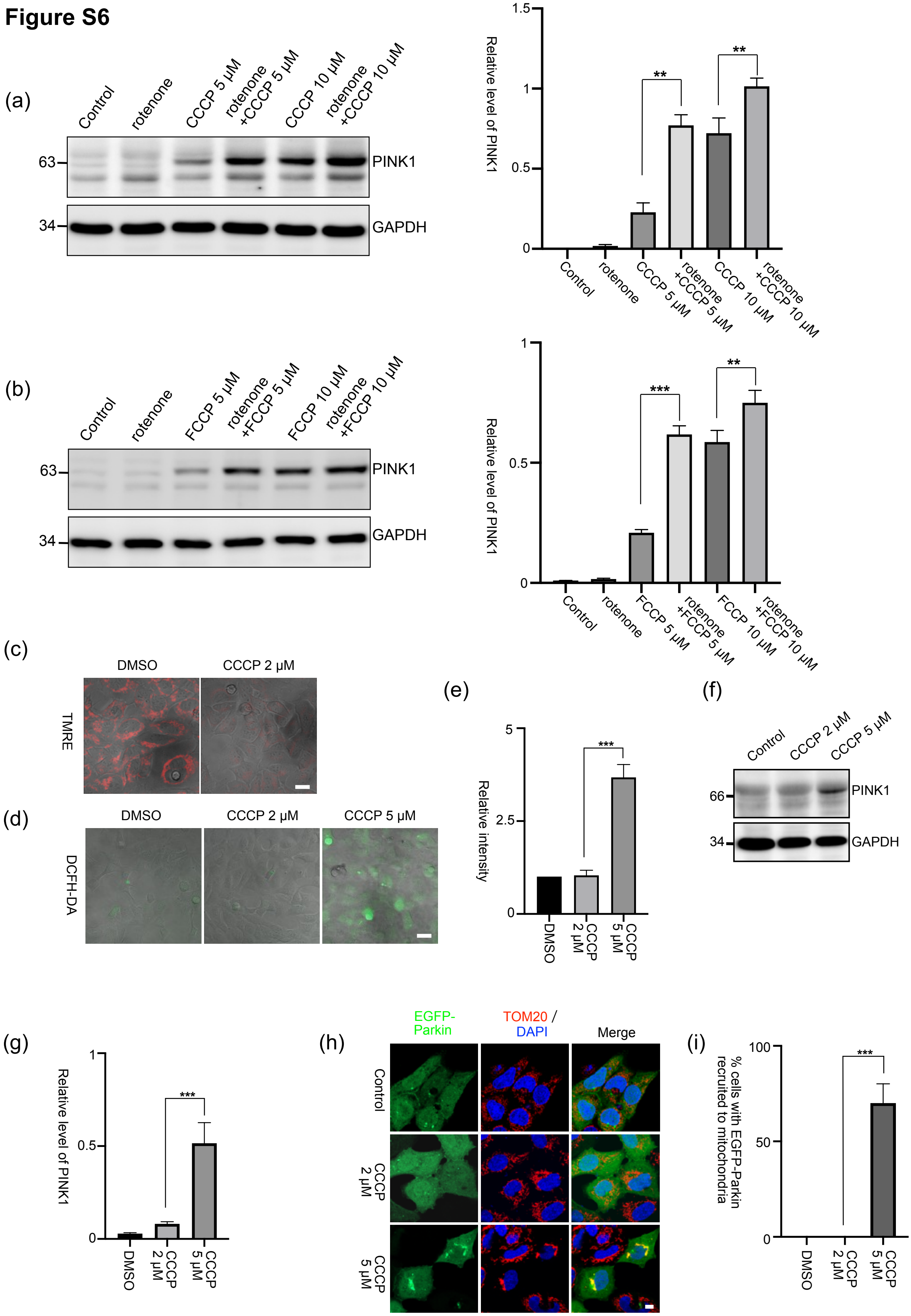
(a)
Control
rotenone
CCCP 5 μM
rotenone +CCCP 5 μM
CCCP 10 μM
rotenone +CCCP 10 μM
63
34
PINK1
GAPDH
Relative level of PINK1
1.5
1
0.5
0
**
**
(b)
FCCP 5 μM
rotenone +FCCP 5 μM
FCCP 10 μM
rotenone +FCCP 10 μM
***
(c)
DMSO
CCCP 2 μM
TMRE
(d)
CCCP 5 μM
DCFH-DA
(e)
Relative intensity
5
2.5
(f)
66
(g)
(h)
EGFP-Parkin
TOM20 / DAPI
Merge
(i)
% cells with EGFP-Parkin recruited to mitochondria
100
50

Figure S6 continue

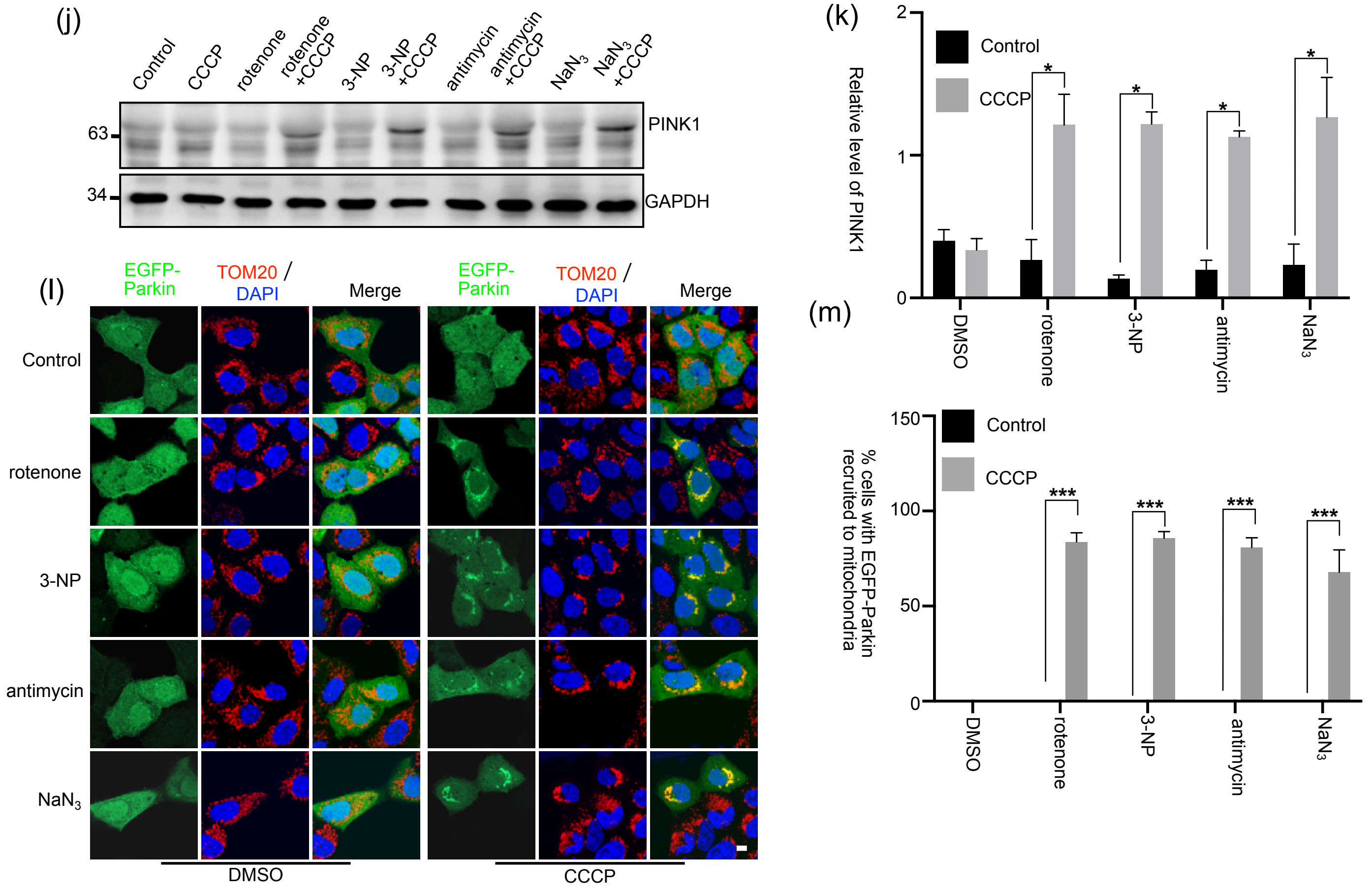

Figure S6. Mitochondrial oxidative stress promoted PINK1 accumulation.

(a and b) HEK293 cells were treated with rotenone (1 μM) for 2 h followed by CCCP (5 or 10 μM) (a) or FCCP (5 or 10 μM) (b) for 1.5 h. The cells were lysed for PINK1 detection. CCCP induced PINK1 accumulation in a dose-dependent manner. However, in combination with rotenone, PINK1 accumulation was significantly increased, suggesting that rotenone-induced ROS generation promotes PINK1 accumulation. Quantitative data are shown as relative density of PINK1 to GAPDH. Mean ± SEM, ** $P < 0.01$, *** $P < 0.001$ by one-way ANOVA.

(c) HEK293 cells were treated with CCCP (2 μM) for 3 h and then stained with TMRE. The results showed that a lower dose of CCCP effectively induced ΔΨm loss.Scale bar, 20 μm.

(d and e) HEK293 cells were treated with 2 μM or 5 μM CCCP for 3 h. The cells were then stained with DCFH-DA for 20 min. (e) Quantitative data are shown as relative fluorescence intensity compared with control, 3 replicates for each group. Mean ± SEM, *** $P < 0.001$ by one-way ANOVA. Scale bar, 20 μm.

(f and g) HEK293 cells were treated with 2 μM or 5 μM CCCP for 3 h. The results showed that a lower dose of CCCP did not induce PINK1 accumulation. Quantitative data are shown as relative density of PINK1 to GAPDH. Mean ± SEM, *** $P < 0.001$ by one-way ANOVA.

(h and i) HEK293 cells were transfected with EGFP-Parkin for 24 h and then treated with CCCP (2 μM) or (5 μM) for 3 h. The results showed that a lower dose of CCCP did not induce Parkin recruitment onto mitochondria. (i) The percentage of cells with EGFP-Parkin recruited to mitochondria from h was quantified, 3 replicates for each group, with >80 cells counted for each replicate. Mean ± SEM, *** $P < 0.001$ by one-way ANOVA. Scale bar, 20 μm.

(j and k) HEK293 cells were pretreated with rotenone (1 μM), 3-NP (10 mM), antimycin (10 μM), or NaN_3 (5 mM) for 2 h and then treated with CCCP (2 μM) or not for 3 h. A lower dose of CCCP did not induced PINK1 accumulation, however, in combination with mitochondrial respiratory chain complex inhibitors, PINK1 was accumulated, further suggesting that mitochondrial respiratory chain complex inhibition-induced ROS generation is necessary for PINK1 accumulation under ΔΨm loss. Quantitative data are shown as relative density of PINK1 to GAPDH (k). Mean ± SEM, * $P < 0.05$ by two-way ANOVA.

(l and m) HEK293 cells were transfected with EGFP-Parkin for 24 h. The cells were then treated with rotenone (1 μM), 3-NP (10 mM), antimycin (10 μM), or NaN_3 (5 mM) for 2 h, followed by CCCP (2 μM) treatment for other 3 h. Consistent with data from (j) that showed an involvement of mitochondrial respiratory chain complex inhibition in PINK1 accumulation upon CCCP treatment, a lower dose of CCCP did not induce Parkin recruitment onto mitochondria unless the cells were pretreated with mitochondrial respiratory chain complex inhibitors. (m) The percentage of cells with EGFP-Parkin recruited to mitochondria from l was quantified, 3 replicates for each group, with >80 cells counted for each replicate. Mean ± SEM, *** $P < 0.001$ by two-way ANOVA. Scale bar, 20 μm.

Figure S7

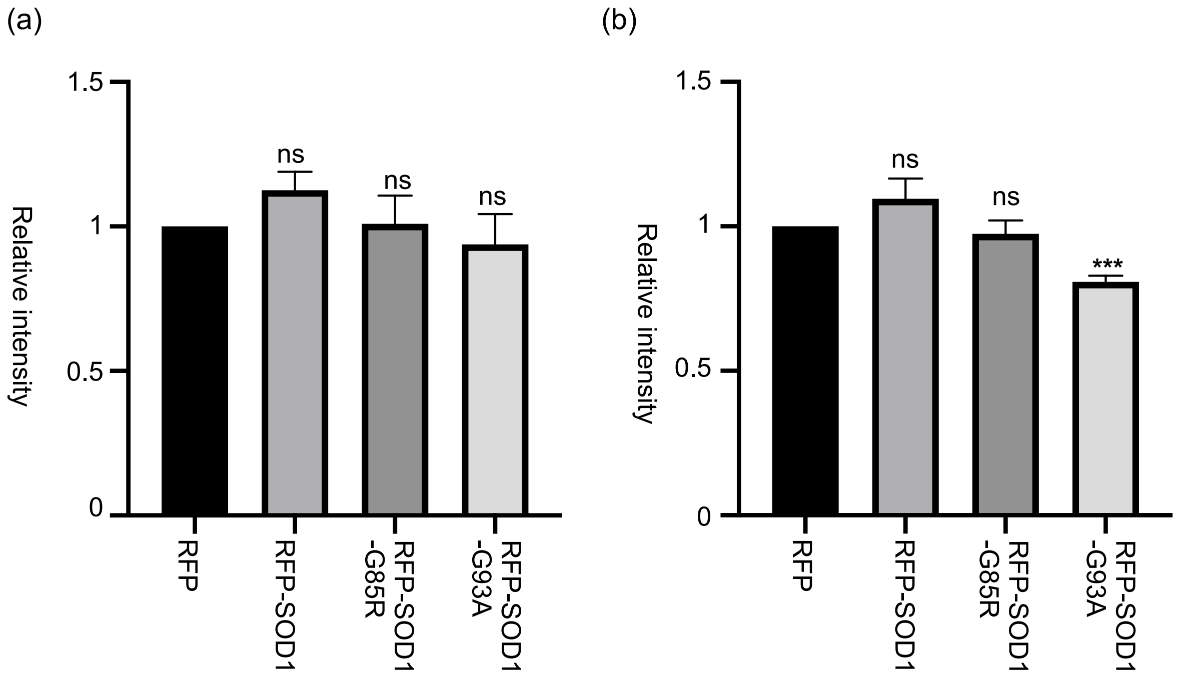

Figure S7. Mitochondrial ΔΨm and ROS generation in HEK293A cells transfected with WT or mutant SOD1

(a) and (b) HEK293 cells were transfected with RFP, RFP-SOD1, RFP-SOD1 G85R or RFP-SOD1 G93A for 48 h. The cells were then stained with DCFH-DA (a) or TMRE (b). Quantitative data are shown as relative fluorescence intensity compared with control, 3 replicates for each group. Mean ± SEM, ns: not significant, *** $P < 0.001$ by one-way ANOVA.
